# A Framework for the Evaluation of Internet-based Diabetes Management

**DOI:** 10.2196/jmir.4.1.e1

**Published:** 2002-01-10

**Authors:** Christian P Mazzi, Michael Kidd

**Affiliations:** ^1^Faculty of MedicineThe University of SydneyChatswood NSW 2067Australia; ^2^Department of General PracticeThe University of SydneyBalmain 2041SydneyAustralia

**Keywords:** Diabetes, Internet, evaluation, therapy, chronic disease management

## Abstract

**Background:**

While still in its infancy, Internet-based diabetes management shows great promise for growth. However, the following aspects must be considered: what are the key metrics for the evaluation of a diabetes management site? how should these sites grow in the future and what services should they offer?

**Objectives:**

To examine the needs of the patient and the health care professional in an Internet-based diabetes management solution and how these needs are translated into services offered.

**Methods:**

An evaluation framework was constructed based on a literature review that identified the requirements for an Internet-based diabetes management solution. The requirements were grouped into 5 categories: Monitoring, Information, Personalization, Communication, and Technology. Two of the market leaders (myDiabetes and LifeMasters) were selected and were evaluated with the framework. The Web sites were evaluated independently by 5 raters using the evaluation framework. All evaluations were performed from November 1, 2001 through December 15, 2001.

**Results:**

The agreement level between raters ranged from 60% to 100%. The multi-rater reliability (kappa) was 0.75 for myDiabetes and 0.65 for LifeMasters, indicating substantial agreement. The results of the evaluations indicate that LifeMasters is a more-complete solution than myDiabetes in all dimensions except Information, where both sites were equivalent. LifeMasters satisfied 32 evaluation criteria while myDiabetes satisfied 24 evaluation criteria, out of a possible 40 in the framework.

**Conclusions:**

The framework is based on the recognition that the management of diabetes via the Internet is based on several integrated dimensions: Monitoring, Information, Personalization, Communication, and Technology. A successful diabetes management system should efficiently integrate all dimensions. The evaluation found that LifeMasters is successful in integrating the health care professional in the management of diabetes and that MyDiabetes is quite effective in providing a communication channel for community creation (however, communication with the health care professional is lacking).

## Introduction

Management of patients with chronic conditions is a long-standing challenge for health care organizations. These conditions include diabetes, chronic heart failure (CHF), chronic obstructive pulmonary disease (COPD), Asthma, HIV/AIDS, and cancer. Patients are required to adopt lifelong exercise, diet, and drug regimens to maintain optimal health and avoid the complications of the disease. These complications can arise suddenly and be life threatening; therefore, patients with chronic diseases must be monitored constantly [1].

In recent years, Internet-based home telemonitoring systems have become available [2]. These sites leverage the Internet to record, measure, monitor, manage, and deliver health care. These information-technology solutions are creating a link between patient and caregiver that enables patients to supply a steady stream of valuable health information to caregivers. For example, diabetics can report their blood glucose readings, thus creating a history of their glucose control, which caregivers can use to evaluate the impact of a therapy (eg, short acting insulin) or the need for a different one [1]. Conversely, caregivers have the ability to provide their patients with crucial information and feedback on the management of their disease. For example, patients can be notified about screening appointments for the complications of diabetes. Therefore, patients benefit from an improved control and understanding of the disease; the ability to self-monitor from home reduces the burden of the disease. These solutions have resulted in dramatic improvements in disease management as measured by hospitalizations [1] and in an overall reduction in costs [3]. Further, patients report higher levels of satisfaction and better control of their conditions [4].

Diabetes is a chronic disease that affects 30 million people worldwide [5] and is the seventh leading cause of death in the United States [6]. The total annual economic cost of diabetes in 1997 was estimated to be US $98 billion. That includes US $44 billion in direct medical and treatment costs and US $54 billion for indirect costs attributed to disability and humanity [7] and a significant intrusion in the life of an individual. In managing diabetes, success is measured by positive change in prognostic indicators and outcomes. Below is a list of measurement criteria used in diabetes management [8,9,10].

- Greater patient self-efficacy
- Greater satisfaction with care, continuity, provider, quality of health outcome
- Decreased HbA _1c_and blood glucose levels
- Improved diet and body weight control
- Lowered cholesterol
- Lowered perception of diabetes intrusiveness
- Improved quality of life
- Less depression
- Decreased incidence of diabetic complications.

Primarily, diabetes must be managed by the patient because it requires adherence to stringent dietary, physical, and medical regimes [8]. Internet-based diabetes management systems have the potential of reducing the burden of disease both to the patient and to the health care system. A recent study found that a high proportion of patients are willing to use Internet resources in the management of their disease [9]. The driving forces behind the proliferation of technology for disease management is the patients' demands to get real-time help, get real-time information, and keep in contact with their physician [1]. Not surprisingly, several diabetes-specific sites have recently appeared [10], including myDiabetes, Health Hero Network, LifeChart, LifeMasters, and Medifor.

The purpose of this paper is to review the patient's and the health care professional's needs in an Internet-based diabetes management solution and to examine how these needs are addressed in practice. An evaluation framework was constructed by grouping the requirements of an Internet-based diabetes management solution into 5 categories: Monitoring, Information, Personalization, Communication, and Technology. Two of the market leaders (myDiabetes and LifeMasters) were selected and evaluated to illustrate the use of the framework.

## Methods

A literature search was conducted on medical databases (Medline, Pre-Medline, EMBASE, Cochrane, and PubMed) and a nonmedical database (Expanded Academic ASAP). The articles were identified by *diabetes*, *chronic disease*, *internet*, and *technology*. The searches were based on the following *AND* combinations of these keywords.

- *diabetes* AND *internet*
- *diabetes* AND *technology*
- *chronic disease* AND *internet*
- *chronic disease* AND *technology*

The exact search methodology differed among databases due to differences in their user interfaces. The methodology for each database is summarized in Table 1.

The abstracts of the articles retrieved by the searches were screened for relevance by the authors. The relevant articles were reviewed in order to compile a comprehensive list of requirements for an Internet-based diabetes management solution. These requirements were identified on the following basis:

- No interdependence between requirements
- Requirements can be assessed as present or not present
- Equal implementation effort required to satisfy the requirements.

The implementation effort was quantified by the number of Use Cases as defined by the Universal Modeling Language (UML) [11,12]. The number of Use Cases ranged from 1 to 3 for each requirement. For example, the requirement defined as *User defined parameter-Patient* allows patients to define which health parameter they wish to monitor. This functionality requires 3 Use Cases: *Identify User,* *Retrieve Parameters*, and *Save Parameters*.

The requirements for Internet-based diabetes management were compiled into the criteria of an evaluation framework. The evaluation criteria were grouped into 5 categories: Monitoring, Information, Personalization, Communication, and Technology. The evaluation framework is presented in Table 2 and the evaluation criteria are discussed in detail in the "Evaluation Criteria" section of the "Results" section.

To illustrate the use of the evaluation framework, we have applied it to 2 existing Internet-based diabetes management systems: my Diabetes (www.myDiabetes.com) and LifeMasters (www.lifemasters.com). These 2 sites were selected because they were first movers in the arena of Internet-based diabetes management. MyDiabetes.com was one of the first sites going live in July 1999, shortly followed by LifeMasters.com in October 1999.

The sites were evaluated from November 1, 2001 through December 15, 2001. The evaluations were performed by 5 independent evaluators who were not aware of each other's ratings. All evaluators are computer literate and are familiar with the use of the Internet. The evaluators included a physician, 3 diabetic patients, and one author [CM]. All the evaluators registered separately with both sites (registration was free). Each evaluator was given a detailed description of the evaluation criteria, as described in the "Results" section, and Table 2, which describes the framework. The evaluators were also given an evaluation form to fill out (effectively Table 3 without results). For each criterion, the evaluators rated the sites as *Yes* if the criterion was satisfied or *No* if it was not satisfied. The evaluations were not supervised.

Figure 1 and Figure 2 are screen shots of the entry forms for the daily glucose measurements forms at myDiabetes and LifeMasters respectively. This basic function of diabetes monitoring requires the user to input his or her blood glucose levels and the time of the readings. The data is stored, effectively creating a log of the glucose control of the patient. LifeMasters records glucose levels based on relative times such as *Bedtime* and asks for symptoms of high and low blood glucose as well as diabetic complications. Mydiabetes records the exact time of the blood glucose measurement but does not screen for any symptoms; this is done in another section of the site.

**Table 1:** Search methodologies for databases

**Database**	**Search Methodology**
Medline (1966 to October week 5, 2001)	*AND* *diabetes* *chronic disease* *internet* *technology* 1 and 3 1 and 4 2 and 3 2 and 4 The 4 terms were searched separately by entering the search string, exploding the subject, and selecting all subheadings. The search results were combined using the AND condition. The search history is described below: diabetes chronic disease internet technology 1 and 3 1 and 4 2 and 3 2 and 4
Cochrane and Pre-Medline	*AND* *diabetes* *chronic disease* *internet* *technology* 1 and 3 1 and 4 2 and 3 2 and 4 The 4 terms were searched separately. The search results were combined using the AND condition. The search history is described below: diabetes chronic disease internet technology 1 and 3 1 and 4 2 and 3 2 and 4
EMBASE (via ScienceDirect), Expanded Academic ASAP, PubMed	*AND All Fields All Years* *diabetes* AND *internet* *diabetes* AND *technology* *chronic disease* AND *internet* *chronic disease* AND *technology* The terms were searched in combination using the AND condition. The terms were searched in All Fields and for All Years indexed. diabetes AND internet diabetes AND technology chronic disease AND internet chronic disease AND technology

**Figure 1:** The myDiabetes entry form for the daily glucose measurements

**Figure 2:** The LifeMasters entry form for the daily glucose measurements

**Table 2:** Evaluation framework

**Evaluation Criteria**	**Description**
**Monitoring**	
User defined parameters	
Health care worker	Health care professionals can specify the parameters to monitor
Patient	Patients can specify the parameters to monitor
User defined parameter ranges	
Health care worker	Health care professionals can specify the normal ranges for monitored parameters
Patient	normal ranges for monitored parameters
Automated data collection	Vital data can be downloaded directly from the measurement device (eg, Glucometer)
Alert algorithms to avoid false alarms	
Entry validation	Validation that patient data is not the result of mistyping (eg, Realistic glucose levels)
Screening of symptoms	Determine if changes in vital data is associated to symptoms indicative of an emergency
Patient involvement in alert	Involving the patient in the decision to notify a health care professional
Multidisciplinary approach	
Multiple aspects of disease management monitored	The monitoring is based on a multidisciplinary approach to diabetes
Physical	Monitoring of physical parameters (blood glucose, weight, blood pressure, etc.)
Social	Monitoring of the social aspects of diabetes (stigma, dieting, etc.)
Psychological	Monitoring of psychological aspects of diabetes (depression, loss of motivation, etc.)
Patient access to multiple specialists	Allowing for communication to multiple experts (dietitians, endocrinologists, etc.)
Proactive outreach	
Notification to patients	medications, health care appointments, etc
Notifications to health care professionals	are reminded of screening test and visits
Feedback	
Retrieve and review medical information	Patients can retrieve their medical data to monitor their progress (tabular or graphical format)
Regular Feedback	control of diabetes is administered and stored
	
**Information**	
Quality of information	site should conform to an accepted level of standards
Pull	
Navigation	Navigation should be based on a logical categorization of data
Search	Search Functionality availability
Push	
Notifications	The system should notify its users of newly available information of interest based on their profile (eg. New research)
Newsletter subscription	Users can subscribe to a specific newsletter that is delivered via e-mail of Web browser
	
**Personalization**	
Assessment and feedback	diabetes should be assessed using standard evaluation tools
Collaborative goal setting	management should be clearly specified
Identification of barriers and supports	Using questionnaires to determine each patient's barrier and the appropriate support measures
Follow-up support	Re-iteration of support measures
Construction of personalized management plan	Tailored management plan as a central feature of the site (can be represented as schedules)
Modification of management plan	The ability for users to modify their plans
Language and ethnicity	Multilanguage delivery and culture conscious content
	
**Communication**	
Health professional- patient	
Synchronous	A channel for one-to-one synchronous communication (eg, videoconferencing)
Asynchronous	A channel for one-to-one asynchronous communication (eg, secure email)
Indirect	Technical representation of the health care professional
Community creation	
Chat rooms	Synchronous many-to-many communication channels
Newsgroups / Forums	Asynchronous many-to-many communication channels
Expert moderation	Communication channels are based on the dialogue with an expert
	
**Technology**	
Security	
Authentication	Identification of users (usually username/password)
Encryption	Data transmission security level (eg, 128-bit)
Usability and user-acceptance	Evaluation of usability and user-acceptance (achieved with questionnaires, usage monitoring etc.)
Reliability and availability	Service should be available at all times
Open architecture	on open standard technologies

### Statistical Analysis

Cohen's multi-rater kappa [13,14] was used to evaluate the agreement between raters for the evaluation framework as a whole. The multi-rater kappa was calculated with SPSS statistical software using the mkappasc procedure.

## Results

### Evaluation Criteria

In this section, we describe in detail the evaluation criteria presented in Table 2.

#### Monitoring

Successful patient monitoring is reliant on efficiently extracting the relevant information from a patient without excessive intrusiveness to both patient and health care professional. Several parameters can be monitored; some examples are blood glucose, weight, blood pressure, diet, foot care, smoking, and nutrition [4,15,16]. Health care professionals should be able to designate which parameters they want to monitor and specify the ranges for each patient. The health care professional should be able to indicate which course of action the system should take if the readings are outside the ranges (eg, notification, triage).

Patients should also be able to designate parameters in an effort to improve self-management and goal setting (addressed in the "Personalization" section of "Evaluation Criteria") [17]; these, however, should be in addition to - and clearly differentiated from - the parameters specified by the health care professional. Patient-designated parameters should not be shared with the health care professional unless the patient desires that they be shared.

The degree of intrusiveness is a fundamental consideration when designing a diabetes management system. A major problem with many disease-management programs using information technology is that they try to collect too much data too often [1]. The desire to collect as much data as possible must be balanced with the disruption it may cause in a patient's life [4]. Successful strategies to reduce intrusiveness are based on automatic data gathering such as Glucometers that transmit glucose readings via the Internet and the use of simplified questionnaires for triage and screening. Intrusiveness to the health care provider is also an important consideration. If systems were designed to send alerts each time a patient's blood sugar readings are outside the normal parameters, the result would be many false alarms. Therefore, systems must have processes in place designed to not overwhelm health care professionals. These processes include entry validation, screening with the use of questionnaires, and patient involvement in the decision to launch an alert [1].

Effective patient monitoring is not limited to the collection of health data, it also requires a multidisciplinary approach, proactive outreach, and feedback.

#### Multidisciplinary Approach

The management of diabetes spans multiple medical specialties as evidenced by the use of multidisciplinary diabetes management teams. For example, an endocrinologist will manage medications and glucose levels, a dietitian will design an appropriate diet, and a psychologist will manage the mental aspect of dealing with diabetes. Internet-based diabetes management programs should be based on a multidisciplinary teamwork. This element consistently appears in successful chronic-disease management systems [18]. Patients should have the ability to interact with multiple specialists to manage each facet of their disease and the Internet can provide a communication channel to enhance this interaction. Successful evaluation tools have been created to effectively measure diabetes management outcomes along multiple dimensions (medical, social, psychological, etc.). Some examples of these tools are the Diabetes Quality of Life Measure (DQOL) developed for use in the Diabetes Control and Complications Trial (DCCT) [19] and the SF-36 [20].

#### Proactive Outreach

Proactive outreach and patient tracking are critical success factors for an Internet-based diabetes management system. Proactive outreach consists of notifications sent to patients to take their medication, visit the health care professional, or simply exercise once a day. The benefit of a proactive approach is well documented in the management of other chronic diseases such as chronic heart failure, where increased compliance and monitoring have resulted in a decrease in the number of hospitalizations for cardiovascular diagnoses and hospital days were reduced from 0.6 to 0.2 (P = .09) per patient per year [21]. Proactive outreach also applies to health care professionals. Reminders to physicians of routine testing for patients can be implemented in an Internet-based diabetes management system. A study determined that the use of a diabetes management system increases the likelihood of physicians ordering lipid-profile testing (19%) and retinal exams for their patients [22].

#### Feedback

The role of the patient has become central in the management of chronic disease; therefore, monitoring must integrate the patient [22]. A crucial aspect of patient integration is feedback. Patients must have the ability to review their medical data at anytime. On-line graphical tools can allow patients to visualize their medical information in much the same way a physician would. Feedback also provides a valuable motivational tool that improves compliance [1] and system usage, both of which are linked to an improved outcome in diabetes management [23].

#### Information

The Internet has always served as a source of health information; 70 million of the 110 million American Internet users have searched the Web for health information in the past year. Currently they can choose from 20,000 health care sites with 1,500 more coming on-line each month [24]. A successful Internet-based diabetes management system should be a source of quality information for the patients who use it. The quality of information on the Internet is a source of great debate. The low barriers to publication on the Internet result in the presence of vast amounts of low-quality and inaccurate information. This misinformation or information that is out of date has the potential of misleading and even harming patients. Consequently, independent agencies such as the Health on the Net Foundation [25] were created to certify the content of medical information on the Internet. Information delivery is based on 2 models: pull and push.

#### Pull Model

The pull model relies on the patient retrieving the information he or she seeks. Two pathways are provided to this end. The patient can retrieve documents by navigating through the Web site or can retrieve information with a search engine.

Navigation requires a clearly-defined information structure. This is effectively implemented with a hierarchical structure that users can follow to retrieve information of increasing level of detail. Navigation should be facilitated by a clear on-screen indication of the user's location in the information hierarchy.

Search engines allow users to search for documents based on keywords. Search engine technology is capable of cataloguing documents based on several criteria. In its simplest form, documents will be catalogued based on their text. Therefore, a search will yield all the documents containing the word that was searched for. However, a successful implementation of a search engine will categorize documents based on several criteria such as topic, author, date, and relevance. Users can then use these criteria to refine their searches.

#### Push Model

The push model involves presenting the information to the patient who has opted to receive it. Relevant information could include new research or newly-released drugs for patients who have specified an interest. Interest can be formally expressed by the patient or can be inferred by the system in an effort to personalize the service (see the "Personalization" section of "Evaluation Criteria").

Information delivery in the push model can be implemented in several ways. Patients can be presented with the relevant information upon logging into the system. Alternatively, technologies such as mobile phones and pagers can be used for delivery. A successful Internet-based management system will implement both models of information delivery.

### Personalization

#### Self-management Plan

The management of any chronic disease must be personalized to the individuals, as they are ultimately responsible for its success. Consequently, an Internet-based diabetes management system must allow patients to tailor the intervention to their specific needs. Patients benefit from a proactive approach to their management (in which they are not told what to do) and gain a valuable insight into the management options that may be available to them [17]. Patient involvement and contribution to disease management has demonstrated improved results and compliance [26].

The comprehensive management of diabetes can be based on several models. It is not the purpose of this paper to discuss these management models but rather their successful implementation as Internet-based diabetes management systems. One such model [17] (multilevel social-ecological model for self-management and support for behavior change) was implemented as a physical-activity intervention study [17]. This model is based on the creation of a *personal action plan* that is the result of both the patient's and health professional's requirements [27]. The creation of a personal action plan can be expressed as these self-management action steps: assessment and feedback, collaborative goal setting, identification of barriers and supports, individualized problem solving, follow-up support, and construction of a personal action plan. Glasgow and Bull have identified the strengths and limitations of interactive technologies such as the Internet for Self-Management Action Steps [17]. Nonetheless, a successful implementation of an Internet-based diabetes management system should provide the patient with the ability to navigate through each action step towards the creation of a personal action plan or the equivalent (depending on the disease-management model used).

#### Language and Ethnicity

Piette et al [28] demonstrated that an Automated Telephone Disease Management (ATDM) system produced positive results with an ethnically-diverse diabetic-patient population. Internet-based diabetes systems can reach different ethnicities by offering their services in multiple languages. In some groups where language may be a barrier to medical care, such systems may provide substantial benefits.

Inevitably, this opens the discussion of Internet demographics splitting patients between *haves* and *have-nots*. This is particularly relevant for type II Diabetes where some minority groups are disproportionately affected and have limited access to the Internet. However, the report from the National Telecommunications and Information Administration indicates a rapid change in Internet demographics that is reflective of the general population of the United States [29].

### Communication

#### Communication Between Health Professional and Patient

Most efforts in health care technology focus on assisting the doctor in diagnosing and treating a disease. This approach tends to omit a key component of the health care cycle: the patient. The new trend in medicine favors the inclusion of the patient as an integral part of the healing process. A review of 22 studies by Stewart et al [30] indicated a positive effect of communication on actual patient health outcome such as pain, recovery from symptom, anxiety, functional status, and physiologic measures of blood pressure and blood glucose.

An Internet-based diabetes management system must be a channel of communication between patients and their health care providers. The communication system can follow 3 models: synchronous, asynchronous, and indirect. Synchronous communication allows the patient and health care provider to communicate directly by using teleconferencing or videoconferencing. Traditionally, these services were in the realm of telemedicine [31] where specific technical equipment was installed to allow the communication to happen. However, the advent of multimedia on the Internet does allow for real-time voice-based and image-based communication. Although at its first steps, synchronous communication can be a valuable part of an Internet-based diabetes management system. Equally, the asynchronous communication model is a crucial part of a management system. Simple solutions such as secure text communication between patient and health care provider can be of great benefit in the management of diabetes. A study at the University of Pittsburgh describes a model of asynchronous communication between doctors and patients that reduced some of the differences in communication in terms of expectations, vocabulary used, and other factors [32]. This study was based on a communication system that allowed patients to familiarize themselves with the relevant domain terms at their own pace. The system also allowed physicians to request more information of patients while providing contextual information. This allowed patients to understand the underlying reasons for the questions.

Lastly, the indirect communication model is based on the concept of representation of the health care professional by technology. Such solutions have been implemented using software agents, a form of artificial intelligence that interacts with its environment and reacts to changes. In this case, the agent can interact with the patient and carry out a basic dialogue - and functions as information search and triage [33]. While still experimental, the use of indirect communication in Internet-based diabetes care shows great potential.

#### Community Creation

Community creation is based on a many-to-many communication channel compared with the one-to-one communication that occurs between health care professional and patient. Community support is a fundamental aspect of self-management of disease. Diabetes patients benefit from discussing topics that concern management of the disease, anxiety as to what the future holds, and interpersonal and social relationships.

The Internet can enable the creation of communities based on the same models of synchronous and asynchronous communication models. One study followed a diabetes chat room for 21 months and found that 79% of all respondents rated participation in the chat as having a positive effect on coping with diabetes [34]. Another study established a chat room for adolescents affected by diabetes and moderated by a diabetologist [35]. The results indicated a decrease in HbA _1c_and an improved capacity for self-management. Anonymity undoubtedly favors a greater freedom of expression of individual problems. Community creation and maintenance should be an integral part of any Internet-based management systems. The implementation can be as synchronous chat rooms or as newsgroups where users communicate asynchronously by posting their comments. Further, experts can moderate chat rooms.

#### Technology

The complex network of human and machine relations involved in managing diabetes via an Internet-based system has strong implications for the design of such a service.

#### Security

One of the main concerns with any medical informatics solution is security and privacy of the data. The success of any Internet-based diabetes management system is reliant on the user's trust that the user's data is secure, private, and confidential. This is possible with the recent availability of strong cryptographic tools used for 2 main purposes: authentication and encryption [23].

##### Authentication

Identification of users is a crucial step in gaining access to the system. Users are granted access to data based on their security profile. For example, only the treating physician can modify a specific patient's blood glucose ranges. Therefore, authentication is both the identification of a user (usually with a combination of username and password) and the enforcement of the security profile. Naturally, user identification is required for more-advanced functions like personalization as mentioned earlier.

##### Encryption

All data transmitted between a patient and the system must be secure. Several encryption algorithms exist, with different strengths and speeds. Generally speaking, most Web servers can establish secure communication links using Netscape's Secure Socket Layer (SSL), which is de facto the Internet standard. Recently, 128-bit encryption has been made available worldwide. Any transmission of patient data should be encrypted at the highest level.

#### Usability and User Acceptance

Testing usability and user acceptance is a critical part of any computerized system and should be a continuous process during the life of the system. Typically, evaluation instruments have consisted of on-line questionnaires, on-line commenting (e-mail), telephone interviews, video-based testing, and tracking of system usage [36].

Many physicians believe that the key success factor in managing diabetes is simplicity [1]. Consequently, the implementation of an Internet-based diabetes management system should strive towards simplicity for both patient and health care professional. Internet technologies can be a great supplement but if the implementation is not user-friendly, it can become a real barrier [1]. Although the technology has enormous potential, developers should not lose sight of the real purpose of these systems: to collect small amounts of data rapidly and efficiently. Therefore, an Internet-based diabetes management system will only be successful if implemented with a simple user interface used to collect the minimum amount of data from the patient (thus reducing its intrusiveness).

#### Reliability and Availability

One of the great advantages of the Internet is that it allows users to access systems anytime and from almost anywhere. This results in a need for systems to always be operational, that is, without downtime. Zero downtime (or close to it) requires fault-tolerant systems. Several technical solutions exist both at the software and hardware level. It is outside the scope of this paper to examine all the solutions; however, it is reasonable to expect an Internet-based diabetes management system to not require downtime for maintenance and to have a fault-tolerant hosting environment.

#### Open Platform

Open technologies are based on nonproprietary standards; therefore, a system can be built using technologies from multiple vendors. This is particularly useful for future expansions or medications to accommodate for increased scalability and functionality requirements. An Internet-based diabetes management system should be based on an open platform, particularly for data exchange. Open standards for data representation such as the eXtensible Markup Language (XML) are being adopted by multiple industries. Consequently, a system built using XML will be able to interface with multiple systems and devices. The same system could deliver its services via multiple devices (Internet, mobile phone, handheld computer, etc.) effectively making the Internet open platform the standard.

### Evaluation of 2 Existing Services

To illustrate the use of the evaluation framework, we have applied it to 2 existing Internet-based diabetes management systems: my Diabetes (www.myDiabetes.com) and LifeMasters (www.lifemasters.com).

To produce an overall evaluation, a criterion was considered satisfactory if the majority of the raters evaluated it positively (*Yes* rating). The results of the evaluations were numerically converted by assigning a value of 1 to all positive (*Yes*) ratings and a value of 0 to all negative (*No*) ratings. The results of all the evaluations are compiled in Table 3. The agreement level is reported for each individual criterion. This was calculated by dividing the number of ratings consistent with the overall rating (the majority) by the number of raters. For example, if a criterion was rated satisfactory or unsatisfactory by 4 out of the 5 raters, the criterion has an agreement level of 80% (4/5).

The technology criteria registered the lowest agreement (60%-80%). The different levels of technical expertise of the evaluators may explain this difference. The Personalization criteria also showed lower levels of agreement between evaluators. This is due to the different interpretations of the criteria between evaluators. Personalization remains a difficult dimension to quantify and evaluate. The quality-of-information agreement levels were also low (60%-80%). Both sites displayed the HON code logo and stated that they subscribed to the HONCode principles. However, neither site was HON registered, although - as of December 14, 2001 - LifeMasters was under review process.

The multi-rater kappa for myDiabetes was 0.75 and for LifeMasters was 0.65, indicating a substantial level of agreement as defined by Landis and Koch [37]. There was an important difference between the kappa of MyDiabetes and the kappa of LifeMasters. Further testing is required to clarify the reasons for the difference.

**Table 3:** Evaluation Examples

Evaluation Criteria	myDiabetes.com (Agreement Level)	LifeMasters.com (Agreement Level)
**Monitoring**		
*User defined parameters*		
Health care worker	No (100%)	Yes (100%)
Patient	Yes (100%)	Yes (100%)
*User defined parameter ranges*		
Health care worker	No (100%)	Yes (100%)
Patient	Yes (100%)	Yes (100%)
Automated data collection	No (100%)	No (100%)
*Alert algorithms to avoid false alarms*		
Entry validation	Yes (80%)	Yes (100%)
Screening of symptoms	Yes (100%)	Yes (100%)
Patient involvement in alert	No (100%)	No (100%)
**Multidisciplinary approach**		
*Multiple aspects of disease management monitored*		
Physical	Yes (100%)	Yes (100%)
Social	Yes (100%). Uses DQOL*	Yes (80%). Uses SF-36
Psychological	Yes (100%). Uses DQOL	Yes (80%). Uses SF-36
Patient access to multiple specialists	No (100%)	Yes (80%)
*Proactive outreach*		
Notification to patients	Yes (100%)	Yes (100%)
Notifications to health care professionals	No (100%)	Yes (100%)
*Feedback*		
Retrieve and review medical information	Yes (100%)	Yes (100%)
Regular feedback	Yes (80%)	Yes (80%)
**Information**		
Quality of information	Yes (80%). Uses HON	Yes (60%). Uses HON
*Pull*		
Navigation	Yes (80%). Categorized	Yes (80%). Categorized
Search	Yes (100%)	Yes (100%)
*Push*		
Notifications	Yes (100%)	Yes (100%)
Newsletter subscription	No (100%)	No (100%)
**Personalization**		
Assessment and feedback	Yes (80%)	Yes (100%)
Collaborative goal setting	No (100%)	No (80%)
Identification of barriers and supports	No (100%)	Yes (80%)
Follow-up support	No (100%)	Yes (80%)
Construction of personalized management plan	Yes (80%)	Yes (80%)
Modification of management plan	Yes (100%)	Yes (100%)
Web site personalization	Yes (100%)	Yes (100%)
Language and ethnicity	No (100%)	No (100%)
**Communication**		
*Health professional - patient*		
Synchronous	No (100%)	Yes (80%)
Asynchronous	No (100%)	Yes (80%)
Indirect	No (100%)	No (100%)
*Community creation*		
Chat rooms	Yes (100%)	No (100%)
Newsgroups / Forums	Yes (100%)	Yes (100%)
Expert moderation	Yes (80%)	Yes (80%)
**Technology**		
*Security*		
Authentication	Yes (100%). User and Password	Yes (100%). User and Password
Encryption	Yes (100%). 128-bit	Yes (100%). 128-bit
Usability and user acceptance	Yes (60%). Tested with forums	No (80%). Not actively tested
Reliability and availability	Netscape compatible	Netscape compatible
Open architecture	No (60%). IIS and ASP	No (60%). IIS and ASP
**Total Positive Results**	25 out of 40	32 out of 40

^*^ DQOL = Diabetes Quality of Life Measure

**Figure 3:**
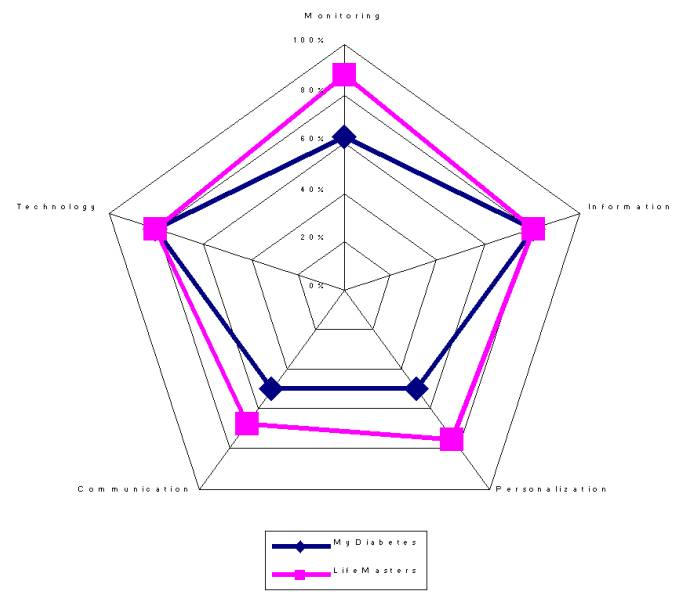
Evaluation of myDiabetes.com and LifeMasters.com. The value of each axis is normalized by conversion to a percentage of the maximum score

#### Graphical Representation

We believe that a graphical representation of the evaluation results is particularly useful for comparing 2 systems and for determining in which direction the systems should expand their services. To this purpose, a radar graph with the 5 axes representing the 5 dimensions of Monitoring, Information, Personalization, Communication, and Technology is a useful representation. The value of each axis is normalized by conversion to a percentage of the maximum score. The evaluation of myDiabetes.com and LifeMasters.com is represented in Figure 3.

The results of the evaluation indicate that LifeMasters is a more-complete solution than myDiabetes in all dimensions - except Information, where both sites were equivalent. This is primarily due to LifeMaster's inclusion of the health care professional in the disease-management cycle. On the other hand, myDiabetes is uniquely interfaced with the patient and is quite good in providing a communication channel for community creation, however, communication with health care professional is lacking, hence the lower score than LifeMasters.

## Discussion

The Internet will undoubtedly change the way we deliver health care services. Chronic disease management, which accounts for 60% of the U.S. medical care costs [38], is a desirable target for the efficiencies of the Internet. Chronic-disease management on the Internet is estimated to have a market potential of US $700 billion [24]. Already we are seeing several Internet-based chronic-disease-management sites arising; however, there is little evidence as to how these solutions are answering the needs of the consumer (the patient).

Consumer health informatics research greatly contributes to the health care sector by attempting to systematize and codify consumer's needs, values, and preferences and by trying to build and evaluate information systems that interact directly with consumers and patients [39]. In this paper, we have attempted to catalogue the critical success factors for an Internet-based diabetes management system based on the available literature and the authors' experience. The result is a first step towards a comprehensive evaluation framework. The framework is based on the recognition that the management of diabetes via the Internet is based on several integrated dimensions, namely, Monitoring, Information, Personalization, Communication, and Technology. A successful diabetes management system should efficiently integrate all dimensions. Therefore, the framework provides a model for evaluation and, more importantly, for strategic growth planning for existing sites. For example, a site that is deficient in the communication dimension may enhance its offerings by adding a synchronous chat room.

This paper reports an initial evaluation of 2 sites. The results indicate a high-level inter-rater agreement as measured by Cohen's multi-rater kappa. However, this is based on a small sample of evaluations (5). Future research should focus on validation of the framework by consistency between larger samples of raters and on correlation with the success of the multiple sites available today. Key metrics for success include the number of enrolled patients; length of time managed; clinical, economic, and quality-of-life outcomes; and patient-satisfaction measures [24].

## Abbreviations

DQOL: Diabetes Quality of Life Measure
XML: eXtensible Markup Language
